# Comprehensive cross cancer analyses reveal mutational signature cancer specificity

**DOI:** 10.1002/qub2.49

**Published:** 2024-06-05

**Authors:** Rui Xin, Limin Jiang, Hui Yu, Fengyao Yan, Jijun Tang, Yan Guo

**Affiliations:** 1 Department of Computer Science, University of South Carolina, Columbia, South Carolina, USA; 2 Department of Public Health and Sciences, Sylvester Comprehensive Cancer Center, University of Miami, Miami, Florida, USA

**Keywords:** cancer specificity, collinearity analysis, DNA mutational signatures, machine learning

## Abstract

Mutational signatures refer to distinct patterns of DNA mutations that occur in a specific context or under certain conditions. It is a powerful tool to describe cancer etiology. We conducted a study to show cancer heterogeneity and cancer specificity from the aspect of mutational signatures through collinearity analysis and machine learning techniques. Through thorough training and independent validation, our results show that while the majority of the mutational signatures are distinct, similarities between certain mutational signature pairs can be observed through both mutation patterns and mutational signature abundance. The observation can potentially assist to determine the etiology of yet elusive mutational signatures. Further analysis using machine learning approaches demonstrated moderate mutational signature cancer specificity. Skin cancer among all cancer types demonstrated the strongest mutational signature specificity.

## 1 | INTRODUCTION

Cancer is a disease in which abnormal cells proliferate without control and invade other tissues. According to the World Health Organization, cancer is a leading cause of death worldwide, accounting for 10 million deaths in 2020. A most primal cause to cancer is somatic mutations, which are acquired during the lifespan of the individual due to risky environmental factors or random errors in DNA replication. Somatic mutations have been studied extensively in cancers [1]. Often somatic mutations are utilized in precision medicine guiding personalized therapeutical choice [2].

Over the last decade, the concept of mutational signature was established and examined thoroughly across multiple cancer types [3, 4]. A mutational signature refers to the characteristic pattern of mutations in a genome attributed to specific biological processes or environmental exposures. These patterns are defined based on relative frequencies of all possible three-nucleotide-long mutation motifs, where each three-base motif consists of a mutation form in the center and accounts for the upstream and downstream of one base neighbors. Since the early attempts of deconvolution of mutational catalogs into mutational signatures, algorithmic improvement and surge of whole-genome sequencing data have catalyzed continual updates of characterized reference signatures. Novel mutational signatures are still yet to be uncovered from the vast, constantly dynamic cancer genomes [5]. Many reference mutational signatures have been associated with particular etiology mechanisms, shedding lights on the underlying causes of DNA damage. Different mutational signatures can be associated with unique types of DNA damage, such as exposure to UV radiation or certain chemicals, or malfunction of specific cellular processes such as DNA replication or repair. By studying these signatures, researchers can gain a better understanding of how somatic mutations arise and how they may contribute to the development of diseases such as cancer.

Cancer heterogeneity is a plaguing topic in oncology that poses challenges for diagnosis, treatment, and monitoring. Cancer heterogeneity can be observed across and within cancer types as different cancer cells may respond differently to therapies or develop resistance to treatment over time. Different cancer types can arise by substantially different mechanisms [6]. A major factor contributing to heterogeneity is somatic mutations that can occur randomly during cell replication or systematically due to exposure to various environmental factors. The nonrandom portion of somatic mutations is captured by the essence of mutational signature. In this study, for the first time, we demonstrate inter- and intra-cancer heterogeneity from the aspect of mutational signature.

## 2 | RESULTS

### 2.1 | Mutational signature and collinearity

**FIGURE 1:**
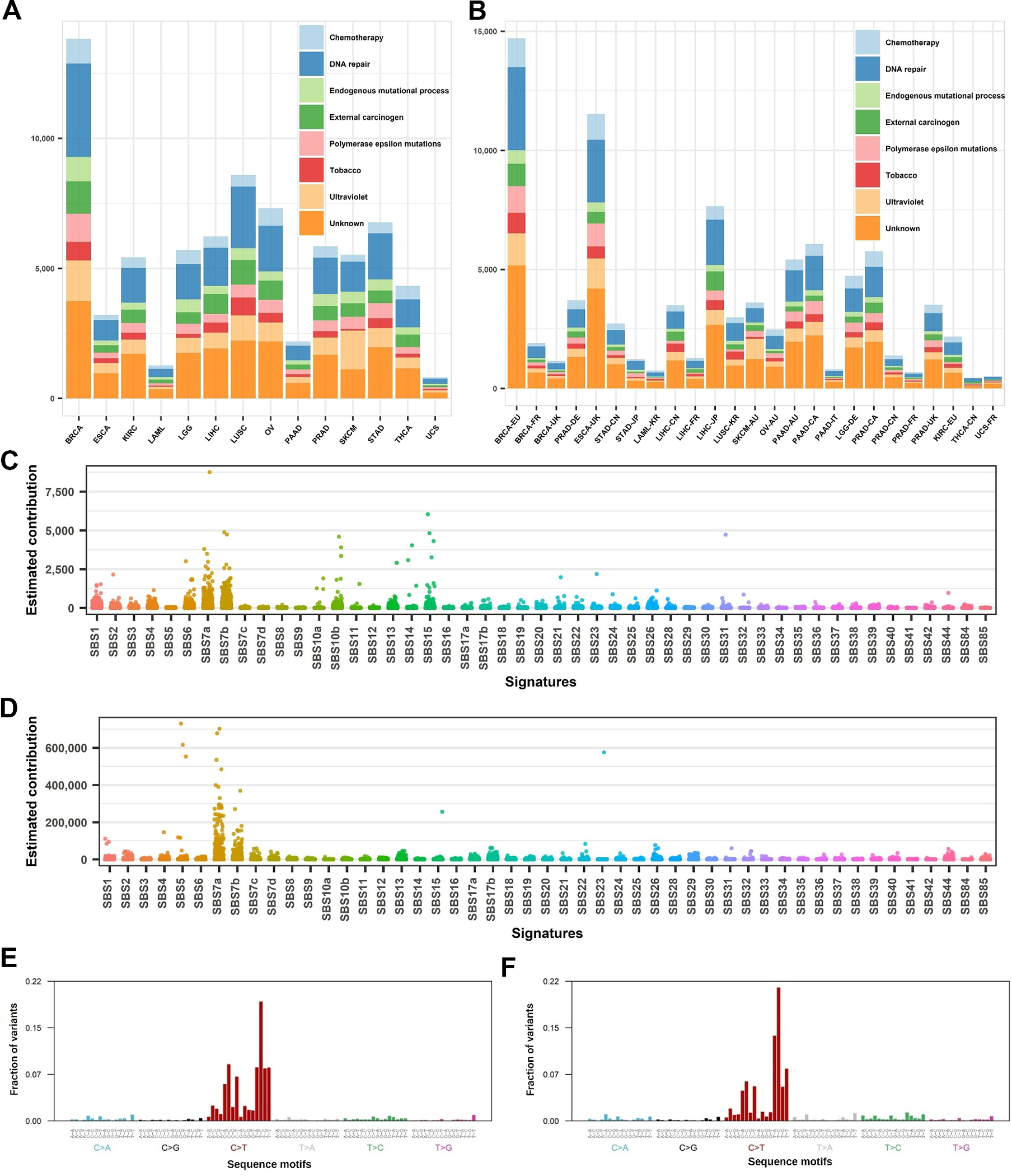
Mutational signature description of the training and testing datasets. (A, B) Dissection of each cancer type by the category of contributing mutational signatures in the training (A) and testing dataset (B). Signature categorization by etiology (different colors) is explained in Supplementary Table S2. (C) Quantitative contributions of each mutational signature to the patients in the training dataset. (D) Quantitative contributions of each mutational signature to the patients in the testing dataset. (E) Overall mutation signature frequency of the 96 three nucleotide-long mutations motifs in TCGA’s skin cancer. (F) Overall mutation signature frequency of the 96 three nucleotide-long mutations motifs in ICGC Australia skin cancer cohort. ICGC, International Cancer Genome Consortium; TCGA, The Cancer Genome Atlas.

We designed a study to evaluate mutational signatures’ cancer specificity by examining mutational signature similarity and by attempting to build machine learning models. The overall training and independent testing datasets including their acronym and sample size are in Supplementary Table S1. The panel of reference mutational signatures and their etiology are in Supplementary Table S2. Mutational signatures were inferred from all mutations and a detailed visual description can be seen in Figure 1. We summarized all mutational signatures into eight major categories (Figure 1A,B) and dissected each cancer type by the categories of contributing mutational signatures (Figure 1C,D). The result of mutational signatures demonstrates cancer etiology. For example, for skin cancer in both training and testing datasets, UV light exposure signatures are the most dominant signature, reflected by characteristic C→T mutations (Figure 1E,F).

**FIGURE 2:**
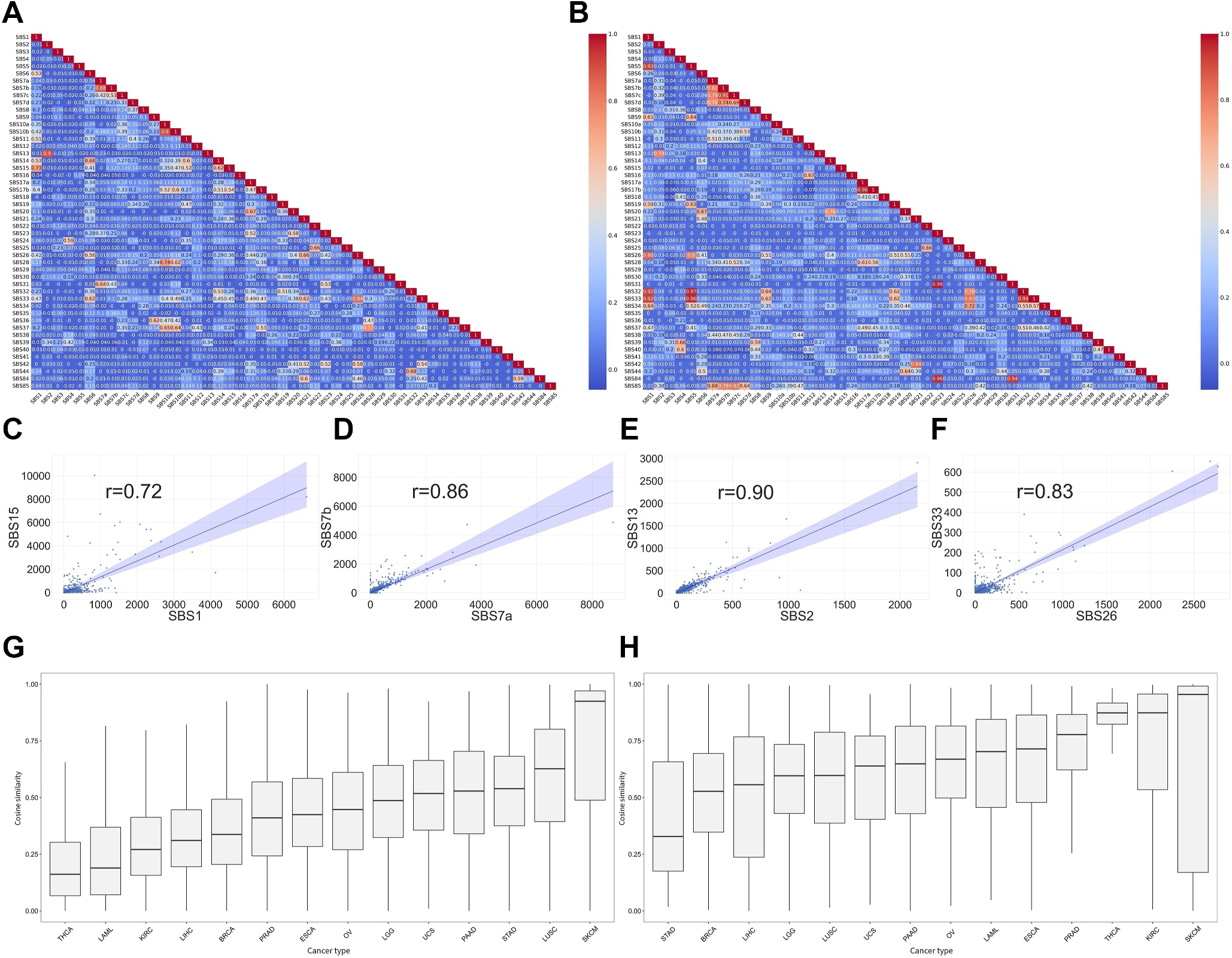
Collinearity analysis results. (A, B) Pearson’s correlation coefficient heatmap between mutational signatures in the training (A) and testing (B) datasets, respectively. (C–F) Four examples of highly correlated mutational signatures. (G, H) Boxplot of pairwise cosine similarity at sample level, computed from mutational signature abundance level for the training (G) and testing (H) datasets, respectively.

We tested whether collinearity exists among the features using Pearson’s correlation coefficient. Figure 2A,B show the correlation matrix heatmap between mutational signatures from SBS1 to SBS85 for the training and testing datasets, respectively. The results show that the majority of mutational signatures are mutually independent, consistent with the assumption that each mutational signature represents a unique cancer etiology. However, there are seven highly correlated (*r* > 0.7) mutational signatures in the training dataset and 20 highly correlated mutational signatures in the testing dataset (Table 1). Three of them are caused by similar etiologies. For example, SBS7a and SBS7b (*r* = 0.88) signatures stem from UV light exposure, as indicated by their mutational signature naming convention. Thus, high similarity between SBS7a and SBS7b is not surprising. Another interesting example is the pair of SBS2 and SBS13 (*r* = 0.9), where both signatures are related to the APOBEC activity. However, SBS2 is represented by T[C→T]A, T[C→T]C, T[C→T]G, and T[C→T]T mutations, whereas SBS13 is represented by a different set of mutations such as T[C→G]A, T[C→G]T, etc. Some correlated signatures, such as the pair of SBS1 and SBS15, have distinct etiologies. SBS1 is initiated by spontaneous or enzymatic deamination of 5-methylacytosine to thymine, which is dominated by mutations A[C→T]G, C[C→T]G, G[C→T]G, and T[C→T]G; SBS15 is associated with DNA mismatch repair and microsatellite instability, which is dominated by several types of C→T mutations and overlaps with SBS1 on several mutation types. In this scenario, the high correlation between SBS1 and SBS15 can be explained by the similarity in mutation pattern. Three of the seven high correlation results involved mutational signatures with unknown etiology. Four exemplar correlated pairs of mutational signatures were plotted for visualization (Figure 2C–F).

Next, we explored sample collinearity based on mutational signatures to illuminate the intrinsic mutational signature similarity of inter and intra cancer types. This analysis was conducted on both The Cancer Genome Atlas (TCGA) and International Cancer Genome Consortium (ICGC) datasets. The results are depicted in Figure 2G,H. Samples in skin cutaneous melanoma (SKCM) consistently had the highest pairwise cosine similarity for both datasets. This suggests that SKCM patients share similar mutation patterns and are thus affected by the same cancer etiology. Cancers with low pairwise cosine similarity such as breast cancer (BRCA) are with more diverse mutational signatures, which may be a result or illustration of the well-known variety of breast cancer subtypes.

### 2.2 | Machine learning

**FIGURE 3:**
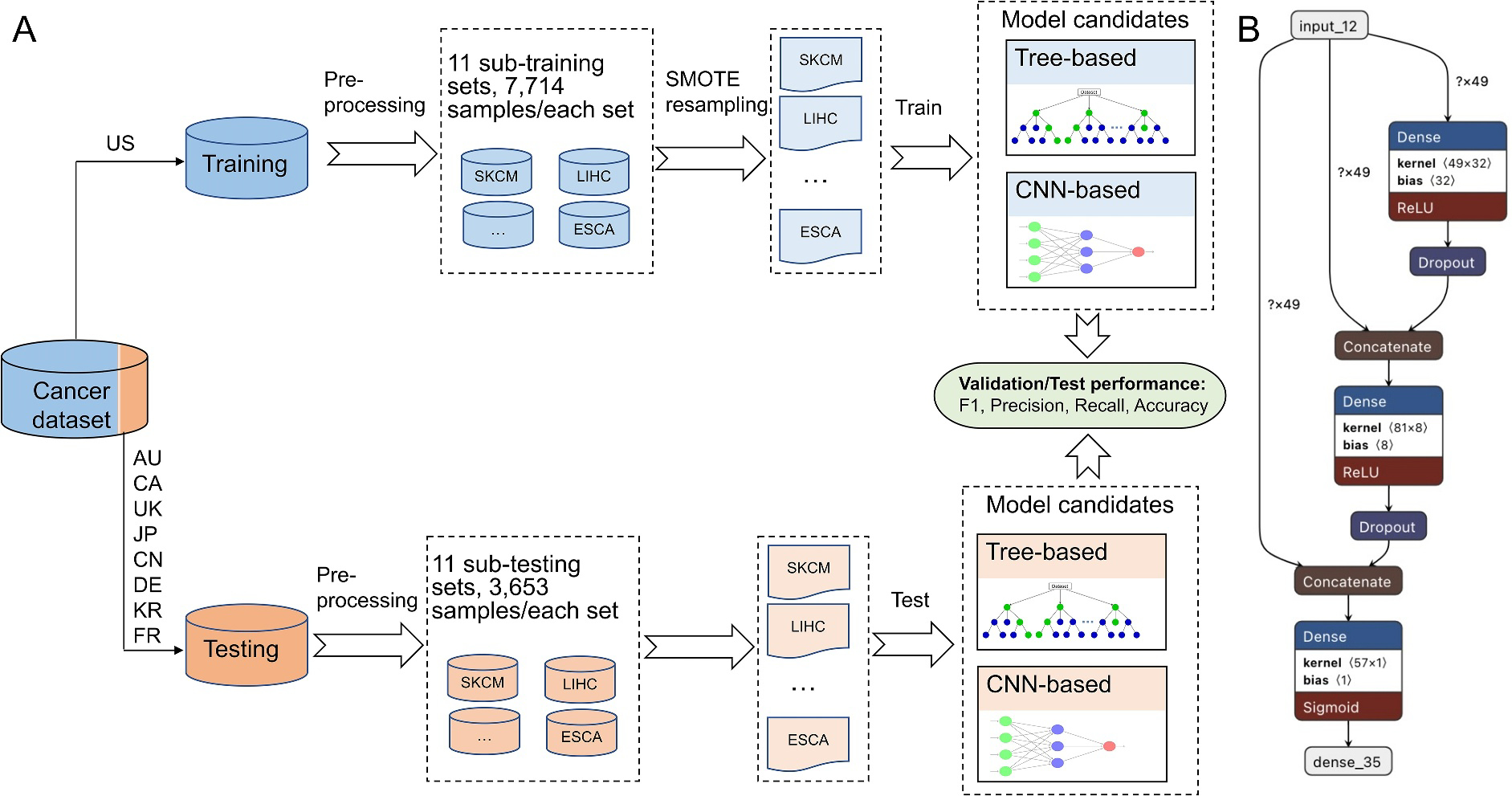
Machine learning study design and deep neural network (DNN) visualization. (A) The study design of machine learning. We utilized training and independent validation to determine if cancer types can be classified by samples’ mutational signature profiles. (B) The structure of the DNN model we applied. AU, Australia; CA, Canada; CN, China; DE, Germany; FR, France; JP, Japan; KR, the Republic of Korea; UK, United Kingdom.

Our machine learning study design, depicted in Figure 3A, makes use of a cancer dataset partitioned into two segments. We employ TCGA data, denoted in blue, as the training dataset. This dataset undergoes preprocessing and rebalancing, details of which are provided in the ‘Mutation data’ chapter’s concluding paragraph. Within this TCGA data, a 20% subset serves as the validation set, contributing to hyperparameter tuning, model selection, and aiding in the prevention of overfitting. The processed training data are then leveraged to train both tree-based and neural network-based models. On the other hand, the ICGC data, represented in orange, functions as an entirely independent testing dataset. This dataset, undergoing preprocessing without rebalancing, is used as an independent metric for assessing the performance of our trained model.

Of note, only exactly matched cancer types (*N* = 11) between the training and testing sets were used in the machine learning portion of this study. To prevent potential patient overlap between the training and testing datasets, all US-based cohorts were removed from the testing dataset. Figure 3B shows one of the structures of the deep neural network (DNN) models utilized in our analysis.

To test mutational signature’s cancer specificity, we deployed five machine learning models, including two tree-based (random forest and XGBoost), two neural network-based (multi-layer perceptron (MLP) and DNN), and one neural architecture search-based (NAS). F1 score was used as the major performance metric. A model was trained for each cancer type by each method. The performance of the models on the independent test dataset is presented in Supplementary Tables S3–S5. The seven top-performing models and their evaluation performance, as shown in Table 2, indicate that models identified through design space exploration, guided by Bayesian optimization, surpass the performance of manually designed neural networks. Among all cancer types, SKCM showed the highest classification performance with a precision of 0.99, recall of 0.8, F1 score of 0.88, an accuracy of 0.99, and an area under curve (AUC) of 0.9. We also discovered that the precision, recall, F1 score, and AUC for Japanese liver cancer cohort (LIHC-JP) were 0.9, 0.8, 0.85, and 0.9, respectively. Additionally, the esophageal adenocarcinoma cohort (ESAD-UK) in the United Kingdom and Renal Cell Cancer (RECA-EU) in European Union also achieved acceptable modeling performance. SKCM is the only cancer type that can be consistently distinguished from other cancer types by mutational signature. A total of 32 models’ F1 scores are significantly higher than the random F1 scores’ negative binomial background distribution, showing that while these models’ performance may not be suitable for consistent and accurate prediction, mutational signatures of these cancer types do show some degree of cancer specificity (Table 3).

**FIGURE 4:**
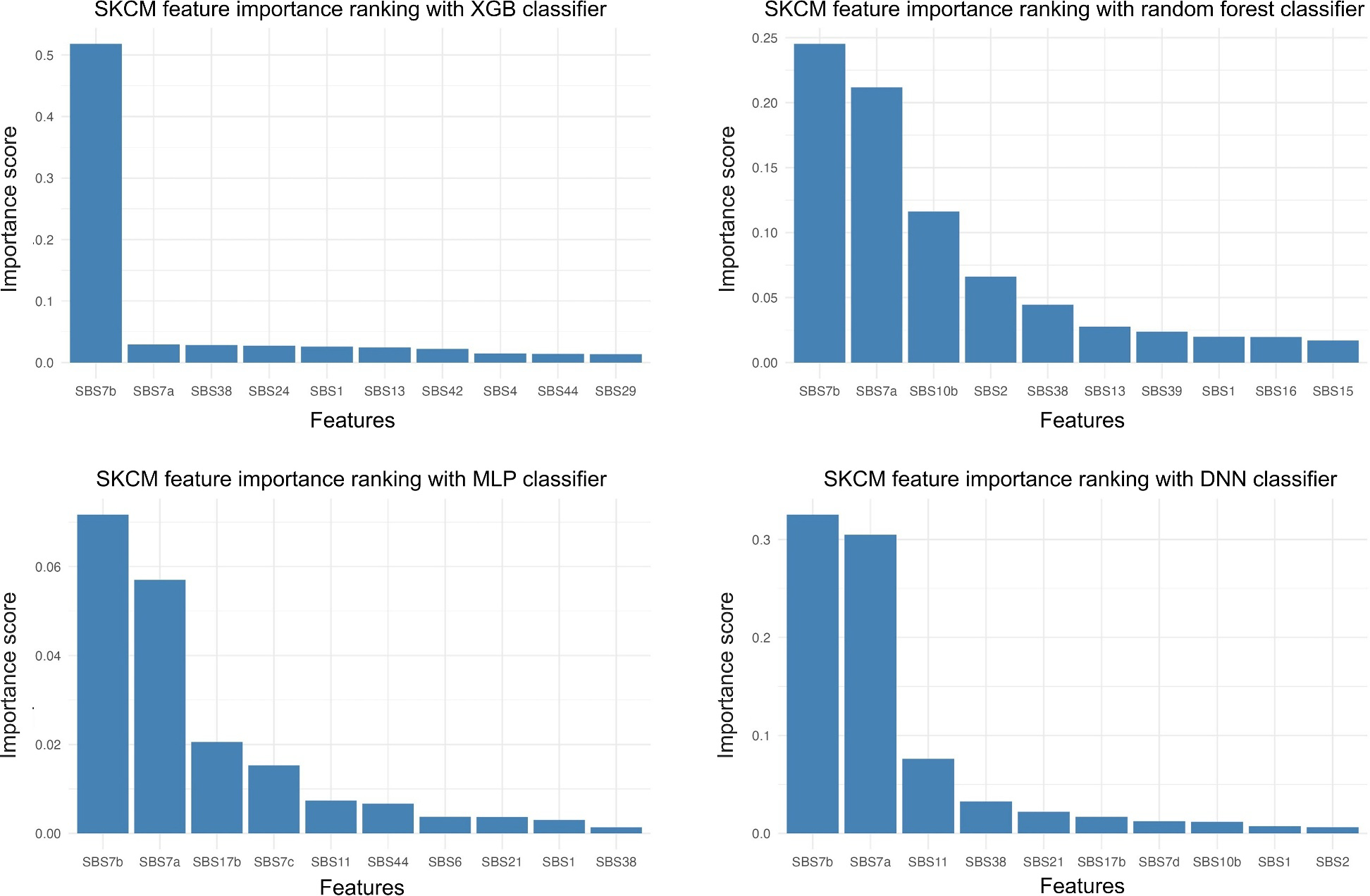
Feature importance ranking by model. XGB, XGBoost.

Next, we ranked the importance of mutational signature based on the SKCM models (Figure 4). The top-10 feature importance scores generated by all models recognized SBS7b and SBS7a as the most important mutational signatures. Both SBS7a and SBS7b are results of UV light exposure, which is a well-known carcinogen for skin cancer. Additionally, all four models’ top-10 features include SBS38 and SBS1. SBS1 is related to spontaneous deamination of 5-methylcytosine, which can be observed in other cancer types. SBS38 is a unique signature only found in skin cancer. The strong combined effect of SBS7 and SBS38 makes SKCM an outstanding cancer type in terms of mutational signature.

## 3 | DISCUSSION

Cancer is a highly heterogeneous disease, and understanding cancer heterogeneity can provide valuable insights into the biology of cancer and the development of more personalized and effective treatments. Cancer heterogeneity can be observed through mutational signatures that reveal diverse mutational processes driving cancer development and progression. In this study, we examined the similarities and uniqueness of mutational signatures across cancer types. Similarities between mutational signatures were examined through collinearity analysis. While the majority of the mutation signatures possess distinct patterns, high correlation between a small portion of mutation signatures indicates that they share redundant mutation patterns.

Of the nearly 100 identified SBS mutational signatures, many are of unknown etiology. By analyzing mutational signature similarity, we show that mutational signatures with substantially different mutation patterns can originate from the same etiology, as demonstrated by the high correlation between SBS2 and SBS13. It was shown that SBS2 and SBS13 often occur in the same samples [7]. Our results show that in addition to co-occurrence, SBS2 and SBS13 also share similar abundance levels. These results suggest that the unknown etiology mutational signature may be estimated based on both the similarity of the mutation pattern and mutational signature abundance. For example, SBS26’s etiology is defective DNA mismatch repair, and SBS33 is of unknown etiology. They have a high correlation of 0.83; both are dominated by T→C mutations with SBS26 displaying more types of T→C mutation variations than SBS33 when accounting for upstream and downstream nucleotides. Speculatively, the SBS33 may potentially share similar or related etiology with SBS26.

During this study, we found that while TCGA and ICGC do share similar mutational signatures for the same cancer types, some differences can be observed. Compared to TCGA, ICGC demonstrates overall higher collinearity between mutational signatures and higher collinearity between samples. The difference between TCGA and ICGC may be due to the type of sequencing data collected. TCGA utilized exome sequencing and ICGC conducted whole genome sequencing. While mutational signature inferred exome sequencing is a good sample of genome-wide mutation spectrum, whole genome sequencing still provides the most accurate mutational signatures. These differences are observable but do not prevent us from concluding that most mutational signatures show little collinearity and that skin cancer possesses the most unique mutational signatures. The existing collinearity may be used to estimate etiology of yet unelucidated mutational signatures. Furthermore, all analyses were performed based on the current understanding of mutational signatures. As a rapidly advancing research field, a mutational signature may receive a refined etiology definition, and novel mutational signatures may be identified. What we have identified in this study contributes to a better understanding of mutational signatures from the collinearity aspects and demonstrates moderate mutational signature cancer specificity.

Mutational signatures have been a hotspot in cancer genomics in the past decade, and all related facets surrounding mutational signatures have been rapidly evolving and updated. Non-negative matrix factorization (NMF), principal component analysis (PCA), and other algorithms were proposed to decompose mutational catalogs into varied contributions of diverse mutational signatures; a plethora of tools, including MutationalPatterns [8], SigProfilerExtractor [5], and others [9] are existent to facilitate all kinds of whole-genome sequencing data mining as relevant to mutational signatures. The present study fills in a tiny gap in the busy research field through two novel approaches. First, the collinearity (similarity) analyses from two orthogonal dimensions, signature-wise and patient-wise, illuminated alternative/distinct angles of etiology association among reference signatures (Figure 2A–F), and drew attention to heterogeneity of signature composition in a cancer cohort (Figure 2G,H). Second, we demonstrated that an array of machine learning models, conventional or modern, intuitive or complex, all can achieve satisfactory classification accuracy for dichotomized prediction of signature contribution. Our research approaches and the resultant observations should inspire future studies of more exquisite designs to pursue deeper in the interesting directions; the present study and future studies in the same direction can hopefully lead to an advanced mechanistic interpretation of reference signatures and guide clinically meaningful stratification of cancer patients per refined characterization of mutational signatures.

Acknowledging the limitations of our study is crucial, and we would like to highlight three primary constraints we encountered.

### Data imbalance:

Our dataset suffers from an imbalance issue, in which there are in sufficient instances of the minority class for the model to effectively learn the decision boundary. To mitigate this, we have used synthetic minority over-sampling technique (SMOTE) to resample the training set, or in other words, synthesize new examples from the minority (positive) class and add them to the training dataset. However, a potential issue arising from this approach is that the generated samples may share too much similarity with the original samples and not be informative enough, potentially leading to overfitting.

### Dataset size:

The size of our dataset may not be large enough for the models to adequately learn the underlying patterns. This could lead to underfitting, a condition where the model may not capture the complexity of the data and thus perform poorly.

### Feature representativeness:

Regardless of the dataset size, there is a possibility that the current features may not be representative or informative enough to train a robust model. This could limit the model’s ability to generalize to new data.

## 4 | MATERIALS AND METHODS

### 4.1 | Collection and preprocessing of mutation data and reference signatures

Somatic mutation data were obtained from TCGA and ICGC. TCGA data were used as training data, and ICGC data were used as independent testing data. After preprocessing, TCGA data involved manageable mutation data for 9096 cancer patients. Profile characterization for 49 reference mutational signatures were downloaded from Cosmic (v2), where each signature had a quantitative profile over 96 trinucleotide motifs and each centered on a central single-base-substitution mutation. The 49 reference signatures were the primary research results of a recent milestone study in the field [3].

To deconvolute mutational signatures, we formalized a catalog of 96 three-nucleotide motifs that surrounded the mutational focus (one upstream nucleotide + mutation site + one downstream nucleotide site) and derived frequency tables of this motif catalog for each involved patient. We leveraged R package MutationalPatterns [10] to fit the patient mutational motif frequency tables to the reference mutational signatures while requiring the coefficients, that is, signature-to-patient contribution strengths, to be non-negative values. The estimated coefficients came out in the form of a 49-by-9096 matrix of non-negative values, representing quantitative strengths or contribution of each signature to the observed, overall mutation in each patient. During model training, the contribution of a mutational signature for each sample is dichotomized into 0 (signature-to-patient contribution equals 0) and 1 (signature-to-patient contribution greater than 0) where 0 denotes that this mutational signature is not present in this sample, and 1 denotes that this mutational signature is present in this sample.

The training dataset consists of 49 mutational signatures and a single label. The entirety of the data are sourced from hospitals located within the United States. The independent test set encompasses 15 countries and regions and covers 11 types of cancers. It is important to note that entries with a sample size of less than 50 in testing were removed to improve the reliability of the test results. To ensure consistency, the labels of testing samples are denoted using the labels from the corresponding training samples. Furthermore, samples with labels that are not present in either the training or testing datasets are excluded. The resulting summary of the training and testing datasets can be found in Supplementary Table S1.

The initial dataset was raw and required pre-processing. We removed the “donor” column, which only contains donor IDs. Data entries with missing values have been excluded, given their insignificant proportion within the entire dataset. Normalization was performed on the data using the Min–Max scaling technique. This process effectively transforms and adjusts data points to conform to a consistent scale between 0 and 1. We also removed categorical features since they were not present in the test set. To create the training and testing datasets, we split the pre-processed dataset in a stratified manner, ensuring that the proportions of cancer samples in the training and testing datasets are the same. Additionally, we utilized the SMOTE [11] over-sampling method to balance the minority class. The SMOTE is employed to balance our dataset. In essence, this process involves choosing a minority class instance and identifying its nearest neighbors within the same class. It then synthesizes new instances that share similarities with these neighbors, thereby increasing the representation of the minority class without mere duplication. This approach allows us to enrich our minority class data, fostering a more balanced and representative dataset for model learning.

### 4.2 | Collinearity analysis

Two types of collinearity analyses were carried out. First, we measured Pearson’s correlation coefficient between any two pairs of mutational signatures. Two mutational signatures were considered highly correlated if Pearson’s correlation coefficient was greater than 0.7. The collinearity analysis of mutational signatures was conducted at the global level by combining all cancer types together. To measure the pairwise correlation between any two samples, we deployed the cosine similarity method. The collinearity analysis of samples was performed by cancer type.

### 4.3 | Decision-tree-based model

The random forest classifier [12] creates a set of decision trees from a randomly selected subset of the training set, and subsequently aggregate the votes from the individual trees to yield the final prediction. The parameter of the number of candidate features at each branching node was set as the square root value of the original feature number. The number of trees in our forest was preset as 100, with no restrictions imposed on the maximum depth or number of leaf nodes for each tree. The growth of trees was guided by the Gini Impurity measure.

The gradient boosted trees approach is widely used and well implemented in the open-source software XGBoost [13]. Gradient boosting is a supervised learning process that combines the predictions of several weaker, simpler models to predict a target variable more accurately. Gradient boosting uses gradient boosting algorithms to iteratively train a series of decision trees. In each iteration, the model calculates the negative gradient of the loss function with respect to the predictions and constructs a decision tree based on the negative gradient. The features that minimize the loss function are used to construct the decision tree, and the predictions from the tree are added to the current model. XGBoost incorporates various optimizations to enhance the algorithm’s efficiency and accuracy. One optimization is the use of a second-order approximation of the loss function, which enables more precise and efficient computation of the gradient. Another optimization involves the use of a regularized objective function to prevent overfitting and improve model generalization. XGBoost also employs diverse techniques to parallelize computation and optimize memory usage, thus empowering itself to handle large datasets of high dimensionality.

### 4.4 | Neural-network-based model

MLP is a popular feedforward neural network used for classification tasks in machine learning. It comprises multiple layers of neurons that apply nonlinear activation functions to input data, ultimately producing a predicted class label. Its ability to handle high-dimensional input data and large datasets makes it a flexible algorithm that can be customized by adjusting various hyperparameters. In our study, we used a specific MLP that employed the limited-memory BFGS solver, a quasi-Newton optimization algorithm suitable for medium-sized datasets. The sizes of hidden layers were set to 32 (1st) and 16 (2nd), thus creating a two-layer neural network with 32 neurons in the first hidden layer and 16 neurons in the second hidden layer. To address the issue of potential overfitting, we set the alpha parameter to 1 × 10^−4^tocontroltheL2regularizationstrength,thereby preventing overfitting. We referred to the specified MLP classifier as the “MLP model” throughout the paper.

Our DNN is an artificial neural network comprising an input layer, two hidden layers, and an output layer. The input layer takes in the aforementioned 49 features and is connected to the first hidden layer, which contains 32 neurons and utilizes the ReLU activation function. To prevent overfitting, a dropout layer with a rate of 0.2 is applied, and its output is concatenated with the input data to create a skip connection. The second hidden layer has 8 neurons and uses the ReLU activation function, followed by another dropout layer with a rate of 0.2. Its output is concatenated with the input data to create another skip connection. The output layer contains a single neuron with a sigmoid activation function, which produces a probability score between 0 and 1. The model is optimized using the Adam optimizer with a learning rate of 0.0001, and the binary cross-entropy loss function is applied. The accuracy metric is used to assess the model’s performance. Training is performed for 100 epochs using a batch size of 8, and the testing data are used to evaluate the model’s performance and help apply early stopping during training.

### 4.5 | Neural architecture search-based model

In this study, we utilized Bayesian optimization for NAS to develop optimal 1D ResNet models tailored to each of the 11 binary classification tasks, as our dataset comprises 11 distinct sub-datasets. NAS effectively explores the search space Ω of possible architectures by considering a combination of hyperparameters, such as the number of residual blocks $O_{1}$, filters $O_{2}$, kernel sizes $O_{3}$, batch normalization usage $O_{4}$, and more, as illustrated in Formula (1).

(1) $$\Omega =O_{1}\times O_{2}\times \cdots \times O_{n}$$

The design of our cross-layer search space configurations is outlined in Supplementary Table S6. This table indicates that our neural network model is structured with 1 to 3 hidden layers, each containing a neuron count ranging between 8 and 64. Each of these hidden layers is followed by a batch normalization layer, a chosen activation layer (from ReLu, tanh, or sigmoid), and a dropout layer with a rate varying from 0.0 to 0.5. Furthermore, for the second and third hidden layers, there may be skip-connections sourced from the earlier layers.

By employing Bayesian optimization, NAS identifies architecture $\alpha^{*}$ out of search space Ω that maximize generalization performance f(a), as illustrated in Formula (2), often outperforming hand-crafted designs and delivering state-of-the-art results with reduced human effort and expertise.

(2) $$\alpha^{*}={argmax}_{\alpha \in \Omega }[f(\alpha )]$$

The resulting tailored 1D ResNet classifier, referred to as the “NAS model,” enhances generalization performance and lowers overfitting risks compared to off-the-shelf architectures.

### 4.6 | Machine learning models evaluation

The performance of the trained models was evaluated using an independent test dataset. Due to the highly imbalanced nature of both the training and testing datasets, we evaluated the model’s performance using an array of metrics, including precision, recall, F1 score, and area under receiver operating characteristic curve (AUC). F1 score is defined as the harmonic mean of precision and recall, which was considered the primary evaluation metric. Furthermore, we used a permutation method to evaluate whether the obtained F1 score was significantly more than the random background. A similar approach was used to identify the most prognostic gene set [14]. Briefly, an original F1 score can be obtained by evaluating the model when matching the cancer type between training and testing datasets. A background of random F1 score can be generated by evaluating the model when intentionally not matching the cancer type between training and testing datasets. Because random F1 score has a range between 0 and 1, the distribution of F1 score forms a negative binomial distribution. Thus, a *p*-value can be inferred from this random background to show whether the original F1 score is significantly higher than the random background.

## Supplementary Material

supplementary

## Figures and Tables

**TABLE 1:** Correlated mutational signature pairs.

Data	Signature 1	Signature 2	Correlation

TCGA	SBS2	SBS13	0.9
TCGA	SBS10a	SBS10b	0.9
TCGA	SBS7a	SBS7b	0.88
TCGA	SBS26	SBS33	0.84
TCGA	SBS28	SBS37	0.77
TCGA	SBS10a	SBS28	0.74
TCGA	SBS1	SBS15	0.73
ICGC	SBS23	SBS31	0.98
ICGC	SBS5	SBS32	0.97
ICGC	SBS5	SBS33	0.96
ICGC	SBS17a	SBS17b	0.96
ICGC	SBS23	SBS84	0.96
ICGC	SBS31	SBS84	0.94
ICGC	SBS32	SBS33	0.94
ICGC	SBS1	SBS5	0.93
ICGC	SBS1	SBS32	0.92
ICGC	SBS1	SBS33	0.92
ICGC	SBS7b	SBS7c	0.91
ICGC	SBS1	SBS26	0.86
ICGC	SBS21	SBS42	0.84
ICGC	SBS26	SBS33	0.83
ICGC	SBS7a	SBS7b	0.82
ICGC	SBS7c	SBS85	0.82
ICGC	SBS5	SBS26	0.8
ICGC	SBS2	SBS13	0.79
ICGC	SBS7a	SBS7c	0.79
ICGC	SBS7b	SBS85	0.79

**TABLE 2:** Model performance.

Cancer type	Region	Precision	Recall	F1	Accuracy	AUC	#Positive samples	Model

KIRC	EU	0.9	0.58	0.71	0.99	0.79	95	NAS
LIHC	JP	0.81	0.75	0.77	0.96	0.86	288	DNN
LIHC	JP	0.9	0.8	0.85	0.98	0.9	288	NAS
SKCM	AU	0.92	0.77	0.84	0.98	0.9	183	MLP
SKCM	AU	0.99	0.80	0.88	0.99	0.9	183	NAS
SKCM	AU	0.99	0.77	0.86	0.99	0.88	183	DNN
SKCM	AU	0.93	0.56	0.7	0.98	0.94	183	XGB

Abbreviations: AU, Australia; EU, European Union; JP, Japan; XGB, XGBoost.

**TABLE 3:** Models that are significant in negative binomial test.

Cancer	Region	Method	F1	Negative binomial *p*

SKCM	AU	DNN	0.86	5.02E–18
SKCM	AU	MLP	0.84	6.28E–17
LIHC	JP	DNN	0.77	6.09E–14
SKCM	AU	XGB	0.7	9.55E–12
ESCA	UK	DNN	0.69	1.78E–11
KIRC	EU	DNN	0.67	5.89E–11
SKCM	AU	RF	0.6	2.35E–09
LUSC	KR	DNN	0.57	9.43E–09
LIHC	CN	DNN	0.55	2.27E–08
BRCA	EU	DNN	0.52	7.89E–08
STAD	JP	MLP	0.5	1.74E–07
LUSC	KR	MLP	0.5	1.74E–07
ESCA	UK	XGB	0.47	5.41E–07
LIHC	CN	MLP	0.41	4.41E–06
STAD	JP	RF	0.36	2.20E–05
LIHC	JP	MLP	0.32	7.37E–05
BRCA	FR	DNN	0.31	9.88E–05
LUSC	KR	XGB	0.3	1.32E–04
ESCA	UK	MLP	0.3	1.32E–04
OV	AU	DNN	0.28	2.33E–04
STAD	JP	XGB	0.25	5.36E–04
LIHC	CN	XGB	0.24	7.04E–04
BRCA	EU	MLP	0.24	7.04E–04
OV	AU	MLP	0.225	1.05E–03
BRCA	FR	XGB	0.22	1.20E–03
STAD	JP	DNN	0.2	2.04E–03
STAD	CN	DNN	0.19	2.66E–03
STAD	CN	MLP	0.17	4.46E–03
OV	AU	XGB	0.17	4.46E–03
LIHC	JP	XGB	0.13	1.25E–02
BRCA	FR	MLP	0.11	2.08E–02
KIRC	EU	MLP	0.09	3.49E–02

Abbreviations: AU, Australia; CN, China; EU, European Union; FR, France; JP, Japan; KR, the Republic of Korea; UK, United Kingdom; XGB, XGBoost.
