# Supplementary material for: Comprehensive cross cancer analyses reveal mutational signature cancer specificity
Source: Quant Biol. 2024 Jun 5;12(3):245–54. doi: 10.1002/qub2.49 (PMC11824353; doi:10.1002/qub2.49)
Supplement: Supplementary file 1 — Supporting Information S1 [file QUB2-12-245-s001.pdf]

Supplementary Tables

Supplementary Table 1. Data distribution review

Countries /Regions	Region Code	Cancer Type	#Samples	Purpose
United States	US	COAD	397	Training
United States	US	PRAD	495	Training
United States	US	LUAD	515	Training
United States	US	SKCM	467	Training
United States	US	ESCA	184	Training
United States	US	CESC	289	Training
United States	US	UCEC	530	Training
United States	US	UCS	57	Training
United States	US	PAAD	177	Training
United States	US	LAML	143	Training
United States	US	LIHC	364	Training
United States	US	BLCA	412	Training
United States	US	BRCA	985	Training
United States	US	THCA	492	Training
United States	US	LUSC	492	Training
United States	US	OV	435	Training
United States	US	STAD	437	Training
United States	US	LGG	507	Training
United States	US	KIRC	336	Training
Australia	AU	PAAD	206	Testing
Canada	CA	PAAD	220	Testing
United Kingdom	UK	ESCA	404	Testing
Australia	AU	SKCM	183	Testing
China	CN	LIHC	175	Testing
Japan	JP	LIHC	288	Testing

Germany	DE	LGG	202	Testing
South Korea	KR	LUSC	145	Testing
Germany	DE	PRAD	159	Testing
United Kingdom	UK	PRAD	136	Testing
China	CN	PRAD	54	Testing
Canada	CA	PRAD	230	Testing
Australia	AU	OV	93	Testing
China	CN	STAD	111	Testing
Japan	JP	STAD	81	Testing
France	FR	BRCA	72	Testing
European Union	EU	BRCA	564	Testing
European Union	EU	KIRC	95	Testing

Supplementary Table 2. SBS feature introduction

SBS Signature	Proposed Etiology (Mutational process)
SBS1	Spontaneous deamination of 5-methylcytosine (clock-like signature)
SBS2	Activity of APOBEC family of cytidine deaminases
SBS3	Defective homologous recombination DNA damage repair
SBS4	Tobacco smoking
SBS5	Unknown (clock-like signature)
SBS6	Defective DNA mismatch repair
SBS7a	Ultraviolet light exposure
SBS7b	Ultraviolet light exposure
SBS7c	Ultraviolet light exposure
SBS7d	Ultraviolet light exposure
SBS8	Unknown
SBS9	Polimerase eta somatic hypermutation activity
SBS10a	Polymerase epsilon exonuclease domain mutations
SBS10b	Polymerase epsilon exonuclease domain mutations
SBS11	Temozolomide treatment
SBS12	Unknown
SBS13	Activity of APOBEC family of cytidine deaminases
SBS14	Concurrent polymerase epsilon mutation and defective DNA mismatch repair
SBS15	Defective DNA mismatch repair
SBS16	Unknown
SBS17a	Unknown
SBS17b	Unknown
SBS18	Damage by reactive oxygen species
SBS19	Unknown
SBS20	Concurrent POLD1 mutations and defective DNA mismatch repair
SBS21	Defective DNA mismatch repair
SBS22	Aristolochic acid exposure
SBS23	Unknown
SBS24	Aflatoxin exposure
SBS25	Chemotherapy treatment
SBS26	Defective DNA mismatch repair
SBS28	Unknown
SBS29	Tobacco chewing
SBS30	Defective DNA base excision repair due to NTHL1 mutations
SBS31	Platinum chemotherapy treatment
SBS32	Azathioprine treatment
SBS33	Unknown

SBS34	Unknown
SBS35	Platinum chemotherapy treatment
SBS36	Defective DNA base excision repair due to MUTYH mutations
SBS37	Unknown
SBS38	Indirect effect of ultraviolet light
SBS39	Unknown
SBS40	Unknown
SBS41	Unknown
SBS42	Haloalkane exposure
SBS44	Defective DNA mismatch repair
SBS84	Activity of activation-induced cytidine deaminase (AID)
SBS85	Indirect effects of activation-induced cytidine deaminase (AID)

Supplementary Table 3. Decision-tree-based model performance across various regions and types of cancer on independent test

Cancer Type	Region	Precision	Recall	F1	Accuracy	AUC	#Positive samples	Model	Scale?
BRCA	EU	0.43	0.01	0.01	0.84	0.61	564	RF	Yes
BRCA	EU	0.47	0.03	0.05	0.84	0.64	564	XGB	No
BRCA	FR	0.33	0.03	0.05	0.98	0.73	72	RF	Yes
BRCA	FR	0.39	0.15	0.22	0.97	0.72	72	XGB	No
ESCA	UK	0.00	0.00	0.00	0.89	0.31	404	RF	Yes
ESCA	UK	0.37	0.64	0.47	0.84	0.85	404	XGB	Yes
KIRC	EU	0.00	0.00	0.00	0.97	0.42	95	RF	Yes
KIRC	EU	0.00	0.00	0.00	0.97	0.48	95	XGB	Yes
LGG	DE	0.00	0.00	0.00	0.94	0.66	202	RF	Yes
LGG	DE	0.00	0.00	0.00	0.94	0.62	202	XGB	Yes
LIHC	CN	0.00	0.00	0.00	0.95	0.71	175	RF	Yes
LIHC	CN	0.51	0.16	0.24	0.95	0.66	175	XGB	No
LIHC	JP	0.00	0.00	0.00	0.92	0.86	288	RF	Yes
LIHC	JP	0.35	0.08	0.13	0.91	0.83	288	XGB	Yes
LUSC	KR	0.50	0.02	0.04	0.96	0.64	145	RF	Yes
LUSC	KR	0.35	0.27	0.30	0.95	0.74	145	XGB	No
OV	AU	0.00	0.00	0.00	0.97	0.62	93	RF	Yes
OV	AU	0.13	0.23	0.17	0.94	0.77	93	XGB	No
PAAD	CA	0.00	0.00	0.00	0.94	0.73	220	RF	Yes
PAAD	CA	0.00	0.00	0.00	0.94	0.62	220	XGB	Yes
PAAD	AU	0.00	0.00	0.00	0.94	0.65	206	RF	Yes
PAAD	AU	0.00	0.00	0.00	0.94	0.55	206	XGB	Yes
PRAD	CA	0.00	0.00	0.00	0.93	0.76	230	RF	Yes
PRAD	CA	0.00	0.00	0.00	0.93	0.68	230	XGB	Yes
PRAD	CN	0.00	0.00	0.00	0.98	0.88	54	RF	Yes

PRAD	CN	0.00	0.00	0.00	0.98	0.68	54	XGB	Yes
PRAD	DE	0.00	0.00	0.00	0.95	0.71	159	RF	Yes
PRAD	DE	0.00	0.00	0.00	0.95	0.57	159	XGB	Yes
PRAD	UK	0.00	0.00	0.00	0.96	0.80	136	RF	Yes
PRAD	UK	0.00	0.00	0.00	0.96	0.63	136	XGB	Yes
SKCM	AU	0.96	0.44	0.60	0.97	0.93	183	RF	No
SKCM	AU	0.93	0.56	0.70	0.98	0.94	183	XGB	Yes
STAD	CN	0.00	0.00	0.00	0.97	0.57	111	RF	Yes
STAD	CN	0.02	0.02	0.02	0.95	0.71	111	XGB	Yes
STAD	JP	0.95	0.22	0.36	0.98	0.86	81	RF	Yes
STAD	JP	0.36	0.20	0.25	0.97	0.78	81	XGB	No

Supplementary Table 4. NN-based model performance across various regions and types of cancer on independent test dataset

Cancer Type	Region	Precision	Recall	F1	Accuracy	AUC	#Positivesamples	Model	Scale?
BRCA	EU	0.35	0.19	0.24	0.82	0.69	564	MLP	No
BRCA	EU	0.62	0.45	0.52	0.87	0.70	564	DNN	No
BRCA	FR	0.08	0.22	0.11	0.92	0.68	72	MLP	No
BRCA	FR	0.21	0.58	0.31	0.94	0.77	72	DNN	No
ESCA	UK	0.43	0.23	0.30	0.88	0.63	404	MLP	No
ESCA	UK	**0.76**	0.64	**0.69**	0.94	0.81	404	DNN	No
KIRC	EU	0.07	0.11	0.09	0.94	0.57	95	MLP	No
KIRC	EU	**0.93**	0.53	**0.67**	0.99	0.76	95	DNN	Yes
LGG	DE	**0.70**	0.03	0.07	0.95	0.53	202	MLP	No
LGG	DE	0.56	0.02	0.05	0.94	0.51	202	DNN	No
LIHC	CN	0.46	0.38	0.41	0.94	0.71	175	MLP	No
LIHC	CN	0.52	0.59	0.55	0.95	0.78	175	DNN	No
LIHC	JP	0.47	0.24	0.32	0.91	0.62	288	MLP	No
LIHC	JP	**0.81**	0.75	**0.77**	0.96	0.86	288	DNN	Yes
LUSC	KR	**0.71**	0.39	0.50	0.97	0.74	145	MLP	No
LUSC	KR	0.51	0.66	0.57	0.96	0.82	145	DNN	Yes
OV	AU	0.15	0.48	0.22	0.92	0.74	93	MLP	No
OV	AU	0.17	0.73	0.28	0.90	0.82	93	DNN	Yes
PAAD	CA	0.00	0.00	0.00	0.94	0.39	220	MLP	Yes
PAAD	CA	0.00	0.00	0.00	0.94	0.50	220	DNN	Yes
PAAD	AU	**1.00**	0.01	0.02	0.94	0.46	206	MLP	Yes
PAAD	AU	1.00	0.00	0.01	0.94	0.50	206	DNN	Yes
PRAD	CA	0.08	0.00	0.01	0.93	0.50	230	MLP	No
PRAD	CA	0.00	0.00	0.00	0.93	0.50	230	DNN	Yes
PRAD	CN	0.00	0.00	0.00	0.98	0.47	54	MLP	Yes
PRAD	CN	0.00	0.00	0.00	0.98	0.50	54	DNN	Yes

PRAD	DE	0.08	0.01	0.01	0.95	0.52	159	MLP	No
PRAD	DE	0.00	0.00	0.00	0.95	0.50	159	DNN	Yes
PRAD	UK	0.00	0.00	0.00	0.96	0.47	136	MLP	Yes
PRAD	UK	0.00	0.00	0.00	0.96	0.50	136	DNN	Yes
SKCM	AU	**0.92**	0.77	0.84	0.98	0.90	183	MLP	Yes
SKCM	AU	**0.99**	0.77	**0.86**	0.99	0.88	183	DNN	No
STAD	CN	0.20	0.14	0.17	0.96	0.78	111	MLP	Yes
STAD	CN	0.12	0.47	0.19	0.88	0.68	111	DNN	Yes
STAD	JP	0.43	0.59	0.50	0.97	0.98	81	MLP	Yes
STAD	JP	0.12	0.62	0.20	0.88	0.75	81	DNN	Yes

Supplementary Table 5. Neural Architecture Search-based model performance across various regions and types of cancer on independent test dataset

Cancer Type	Region	Precision	Recall	F1	Accuracy	AUC	#Positive samples	Model	Scale?
BRCA	EU	0.53	0.14	0.22	0.84	0.56	564	NAS	Yes
BRCA	FR	0.1	0.11	0.11	0.96	0.54	72	NAS	Yes
ESCA	UK	0.66	0.63	0.64	0.92	0.79	404	NAS	Yes
KIRC	EU	0.9	0.58	0.71	0.99	0.79	95	NAS	No
LGG	DE	0.13	0.02	0.04	0.94	0.51	202	NAS	No
LIHC	CN	0.78	0.52	0.62	0.97	0.76	175	NAS	Yes
LIHC	JP	0.9	0.8	0.85	0.98	0.9	288	NAS	Yes
LUSC	KR	0.64	0.5	0.56	0.97	0.74	145	NAS	Yes
OV	AU	0.23	0.65	0.33	0.93	0.79	93	NAS	Yes
PAAD	CA	0.00	0.00	0.00	0.94	0.50	220	NAS	Yes
PAAD	AU	0.25	0.00	0.01	0.94	0.5	206	NAS	Yes
PRAD	CA	0.5	0.00	0.01	0.93	0.5	230	NAS	No
PRAD	CN	0.00	0.00	0.00	0.98	0.5	54	NAS	No
PRAD	DE	0.00	0.00	0.00	0.95	0.5	159	NAS	No
PRAD	UK	0.00	0.00	0.00	0.96	0.5	136	NAS	No
SKCM	AU	0.99	0.8	0.88	0.99	0.9	183	NAS	No
STAD	CN	0.18	0.06	0.09	0.96	0.53	111	NAS	Yes
STAD	JP	0.64	0.72	0.67	0.98	0.85	81	NAS	Yes

Supplementary Table 6. Cross-layer Search Space

Architecture Options	Range	type
Number of Layers	1-3	discrete
Number of hidden neurons in Layer_x	8-64	discrete
Skip connection from which layer	0,1, …, x-1	discrete
Dropout rate	0.0-0.5	continuous
Activation function	["relu", "tanh", "sigmoid"]	discrete
